# Supplementary material for: Characterization of zebrafish rod and cone photoresponses
Source: Sci Rep. 2025 Apr 18;15:13413. doi: 10.1038/s41598-025-96058-8 (PMC12008237; doi:10.1038/s41598-025-96058-8)
Supplement: Supplementary file 1 — Supplementary Material 1 [file 41598_2025_96058_MOESM1_ESM.docx]

**Supplementary Information**

**Supplemental Table S1** Spectral spread for the wavelengths of test flashes

| Wavelength (nm) | Light source | Bandpass filter | Full width at half maximum (nm) |
| --- | --- | --- | --- |
| 405 | M405L4^a^ | (Not used) | 12.5 |
| 425 |  | #87-787^b^ |  |
| 450 |  | #88-300^b^ |  |
| 475 |  | #87-788^b^ |  |
| 500 |  | #88-300^b^ |  |
| 525 |  | #87-789^b^ |  |
| 550 | MNWHL4^a^ | #88-300^b^ | 25 |
| 575 |  | #87-790^b^ |  |
| 600 |  | #88-300^b^ |  |
| 625 |  | #87-791^b^ |  |
| 650 |  | #88-300^b^ |  |
| 675 |  | #87-792^b^ |  |
| ^a^Thorlabs, ^b^Edmund Optics. | |  |  |

**
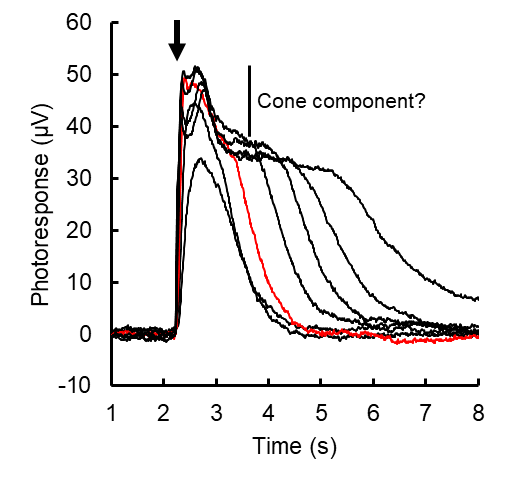
**

**Supplemental Figure S1** Waveform of rod-cone mixed responses after subtracting isolated cone component (Fig. 7c). Flash intensity: 32 to 32000 photons µm^-2^, Red: 320 photons µm^-2^. Timing of the test flash is shown by an arrow. Cone response-like bumps are still observed on the rod response plateau indicating incomplete rod-cone response separation.
